# Herbicide ingredients change *Salmonella enterica* sv. Typhimurium and *Escherichia coli* antibiotic responses

**DOI:** 10.1099/mic.0.000573

**Published:** 2017-11-17

**Authors:** Brigitta Kurenbach, Paddy S. Gibson, Amy M. Hill, Adam S. Bitzer, Mark W. Silby, William Godsoe, Jack A. Heinemann

**Affiliations:** ^1^​ School of Biological Sciences, University of Canterbury, Christchurch, New Zealand; ^2^​ Centre for Integrated Research in Biosafety and Centre for Integrative Ecology, University of Canterbury, Christchurch, New Zealand; ^3^​ Department of Biology, University of Massachusetts Dartmouth, Dartmouth, MA, USA; ^4^​ Bio-Protection Centre, Lincoln University, Lincoln, New Zealand

**Keywords:** pesticides, antibiotics, antibiotic resistant bacteria

## Abstract

Herbicides are frequently released into both rural and urban environments. Commercial herbicide formulations induce adaptive changes in the way bacteria respond to antibiotics. *Salmonella enterica* sv. Typhimurium and *Escherichia coli* were exposed to common co-formulants of formulations, and *S. enterica* sv. Typhimurium was exposed to active ingredients dicamba, 2,4-D and glyphosate to determine what ingredients of the commercial formulations caused this effect. Co-formulants Tween80 and carboxymethyl cellulose induced changes in response, but the pattern of the responses differed from the active ingredients, and effect sizes were smaller. A commercial wetting agent did not affect antibiotic responses. Active ingredients induced changes in antibiotic responses similar to those caused by complete formulations. This occurred at or below recommended application concentrations. Targeted deletion of efflux pump genes largely neutralized the adaptive response in the cases of increased survival in antibiotics, indicating that the biochemistry of induced resistance was the same for formulations and specific ingredients. We found that glyphosate, dicamba, and 2,4-D, as well as co-formulants in commercial herbicides, induced a change in susceptibility of the potentially pathogenic bacteria *E. coli* and *S. enterica* to multiple antibiotics. This was measured using the efficiency of plating (EOP), the relative survival of the bacteria when exposed to herbicide and antibiotic, or just antibiotic, compared to survival on permissive media. This work will help to inform the use of non-medicinal chemical agents that induce changes in antibiotic responses.

## Introduction

The widespread use of antibiotics has led to antibiotic-resistant human pathogens. Multi-drug-resistant pathogens are now a serious but common occurrence complicating treatment, increasing morbidity and mortality, and resulting in increased costs to health systems [1–3]. While mutations and gene acquisition through horizontal gene transfer that lead to changes in the minimum inhibitory concentration (MIC) of antibiotics in clinical settings have received the most attention, microbes in many environments are exposed to antibiotics at sub-inhibitory levels. This can have a wide range of physiological consequences [4]. Sub-inhibitory antibiotic exposures may occur in livestock, feed, soil and areas polluted by environmental releases of antibiotics such as waterways and sewage ponds. Even within patients, antibiotic concentrations vary with time and location in the body, creating ‘grey zones’ where antibiotic concentrations fall to sub-lethal levels. Grey zones can occur, for example, at the end of a treatment course or because of inconsistent adherence to treatment. Small increases in the MIC, either through mutations that incrementally increase resistance or through an adaptive response, extend the range of sub-lethal concentrations and hence the probability of treatment failure [5, 6].

‘Adaptive’ responses to antibiotics that temporarily increase the MIC can be caused by changes in gene expression [7]. These often result in changes to efflux and permeability [8]. Adaptive resistance is initiated by exposure to an environmental trigger. Many bacterial efflux pumps are not substrate-specific, so once induced they confer cross-protection to other toxins [9]. This has been demonstrated for combinations of antibiotics [10], unrelated substances like salicylic acid [11, 12] or bile salts [13], or abiotic stresses from changes to pH or anaerobiosis [8].

Bacteria revert to their previous state of susceptibility after the trigger is removed. Adaptive resistance can complicate treatment because therapeutic doses are determined by MIC measurements *in vitro* under laboratory conditions in the absence of such triggers [14].

We previously added three commercial herbicide formulations to the list of triggers. They caused either increases, decreases, or no change in the survival on antibiotics when bacteria were exposed to herbicides and antibiotics concurrently, compared to only antibiotic exposure. Increases were shown to be additive and consistent with an induction of efflux pumps [15]. Herbicide formulations are complex chemical mixtures of active and inert ingredients. Active ingredients are those intended to confer the pesticidal activity of the product [16]. Inert ingredients are defined by the US Environmental Protection Agency as co-formulants ‘other than an active ingredient that is intentionally included in a pesticide product’ [16]. We attempted to identify the kinds of ingredients that could potentially induce an antibiotic response. We tested two hypotheses: (1) active ingredients and (2) co-formulants induce changes in antibiotic response. The active ingredients of these herbicide formulations, dicamba (3, 6-dichloro-2-methoxybenzoic acid), 2,4-D (2,4-Dichlorophenoxyacetic acid) and glyphosate (*N*-(phosphonomethyl)glycine), and two other compounds commonly used as surfactants identified from patent applications as components of herbicide formulations, Tween80 and carboxymethyl cellulose (CMC) [17, 18], were used to test the hypotheses. Deletion mutant phenotypes and transcriptomics were used to further test the hypothesis that efflux pumps were induced by the commercial formulations Kamba and Roundup.

## Methods

### Bacteria

Bacteria are described in Table 1. JW2454 was purchased from NBRP: *E. coli* (NIG, Shizouka, Japan). Bacteria were cultured at 37 °C using LB Lennox (Invitrogen, Auckland, NZ) medium supplemented as appropriate with ampicillin (Amp; AppliChem, Dunedin, NZ), chloramphenicol (Cam; Sigma, Auckland, NZ), ciprofloxacin (Cip; Pentex, Auckland, NZ), kanamycin (Kan; Gibco, Auckland, NZ) or tetracycline (Tet; Sigma, Auckland, NZ). Liquid cultures were grown with aeration (180 r.p.m.), and plates were incubated in plastic bags to avoid drying out. Commercial herbicide formulations were Kamba^500^ (Nufarm, Otahuhu, NZ) containing 500 g l^−1^ dimethyl salt of dicamba, and Roundup Weedkiller (Monsanto, Melbourne, AU) containing 360 g l^−1^ isopropylamine salt of glyphosate. Active ingredients were dicamba, 2,4-D and glyphosate (Sigma, Auckland, NZ). Co-formulants were Tween80 (BDH, Auckland, NZ), Pulse Penetrant (Yates, Auckland, NZ) containing 800 g l^−1^ organo-modified polydimethyl siloxane, and carboxymethyl cellulose (Sigma, Auckland, NZ). Herbicide concentrations are reported in parts per million acid equivalent (ppm ae) to enable comparison with other formulations.

**Table 1.** Bacteria

Strain	Genotype, comments	Reference
*E. coli*		
BW25113	(Wild-type) F^−^, λ^−^, Δ*(araD-araB)567*, *ΔlacZ4787(*::*rrnB-3), rph-1,* Δ*(rhaD-rhaB)568, hsdR514*	[22]
CR5000	BW25113 Δ*acrB*	[22]
CR7000	BW25113 Δ*acrA*	[39]
JW0912	BW25113 Δ*ompF *:: *kan*, Kan^R^	[22]
JW2454	BW25113 Δ*acrD *:: *kan*, Kan^R^	[22]
JW5503	BW25113 Δ*tolC *:: *kan*, Kan^R^	[22]
*S. enterica* sv. Typhimurium		
SL3770	LT2, *pyr*^+^, *rfa*^+^	[40]

To account for day-to-day differences in the densities of the culture used, results are reported as the efficiency of plating (EOP), the ratio of the titre of a culture on treatment medium to the titre of the same culture on LB medium [(c.f.u. ml^−1^)_treatment_/(c.f.u. ml^−1^)_LB_] [12]. Changes in EOP (antibiotic effectiveness) and concentration of the inducing agent (dose responses) were determined as described previously [15]. In brief, bacteria were cultured to saturation (approx. 2×10^9^ c.f.u. ml^−1^) without selection and dilutions were plated on LB supplemented with antibiotics and/or herbicides/ingredients (see figure legends for concentrations). Plates were examined and colonies counted daily for up to 4 days, at which time no new colonies emerged. Results are the average of at least three independent experiments.

### Statistics

**Fig. 1.**
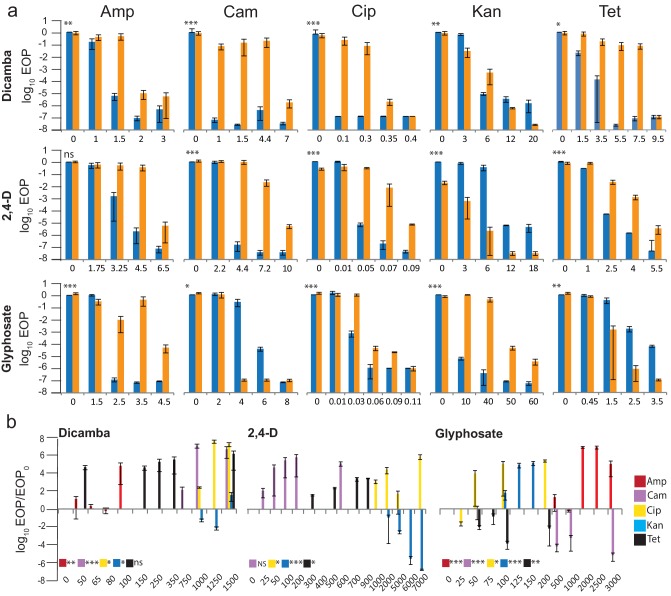
Change in EOP (a) when *S. enterica* is (orange) and is not (blue) exposed to active ingredients. Concentration of active ingredient needed to induce a response (b). (a) The x-axis scale is antibiotic concentrations in µg ml^−1^. Dicamba concentrations were always 1500 ppm ae. For Amp/Cam/Cip/Kan/Tet, respectively, 2,4-D concentrations were na/1500/5000/6000/500 ppm ae and glyphosate were 3000/3000/200/200/3000 ppm ae. (b) The x-axis scale is the concentration of ingredients in ppm ae. For Amp/Cam/Cip/Kan/Tet, respectively, concentrations used were 1.5/4.4/0.1/6/3.5 µg ml^−1^ dicamba, na/4.4/0.05/6/2.5 µg ml^−1^ 2,4-D and 2.5/4/0.05/12/2.5 µg ml^−1^ glyphosate. Values are averages of at least three independent experiments; error bars are sem (standard deviation/√n). Asterisks indicate *P*-values (see Methods). *P<0.05; **P<0.01; ***P<0.001, ns, not significant.

**Fig. 2.**
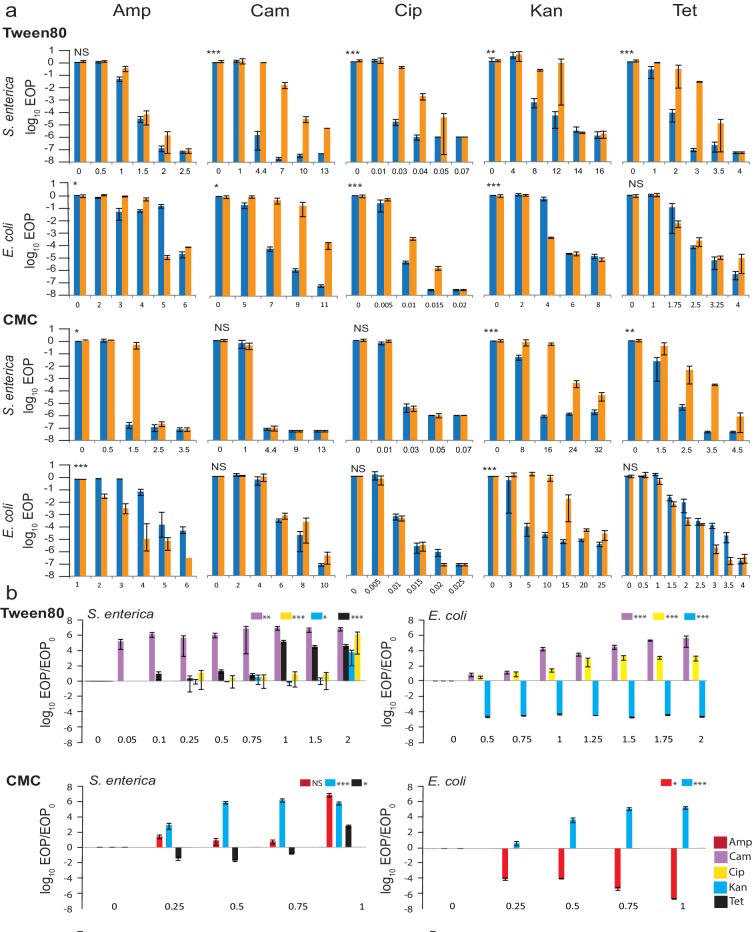
Change in EOP (a) when *S. enterica* is (orange) and is not (blue) exposed to co-formulant. Concentration of co-formulant needed to induce a response (b). (a) The x-axis scale is antibiotic concentrations in µg ml^−1^. Tween80 was used at 2 % (v/v), CMC was used at 1 % (w/v). (b) The x-axis scale is concentration of surfactants in %. *S. enterica*: concentrations of Amp/Cam/Cip/Kan/Tet, respectively, were na/4.4/0.03/12/2 µg ml^−1^ with Tween80 and 1.5/na/na/0.25/2.5 µg ml^−1^ with CMC. *E. coli*: concentrations of Amp/Cam/Cip/Kan/Tet, respectively, were na/7.5/0.01/0.5/na μg ml^–1^ with Tween80 and 5/na/na/10/na μg ml^−1^ with CMC. Values are averages of at least three independent experiments; error bars are sem (standard deviation/√n). Asterisks indicate *P*-values (see Methods). *P<0.05; **P<0.01; ***P<0.001, ns, not significant.

**Fig. 3.**
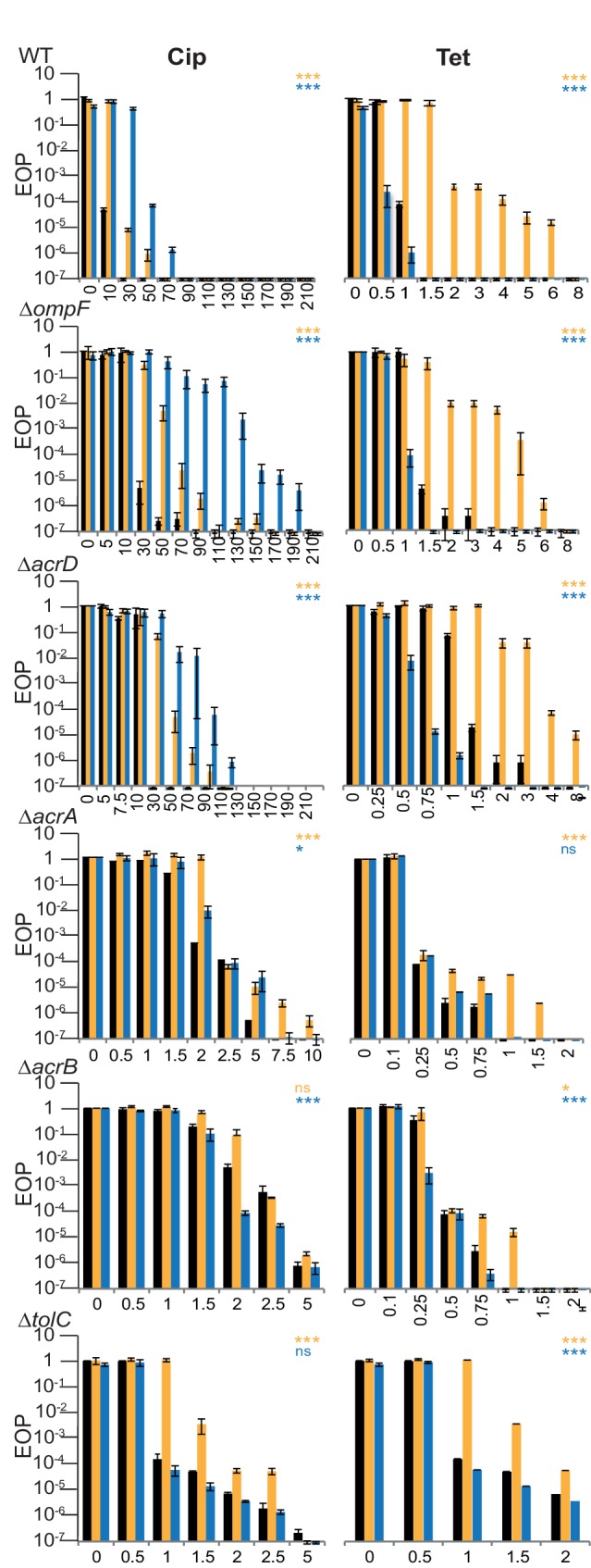
Change in EOP of WT and strains with pump and porin gene deletions with (orange columns Kamba, blue columns Roundup) or without (black columns) added herbicide. The x-axis scale is antibiotic concentration. Left panel: Cip (ng ml^–1^), right panel: Tet (µg ml^–1^). Note that the x-axes were expanded for the Δ*acrA*, Δ*acrB* and Δ*tolC* strains. Herbicide concentrations were 1380 ppm ae Kamba for all strains, 1240 ppm ae Roundup for WT, *ΔompF*,and *ΔacrD*, and 25 ppm ae for Δ*acrA,* Δ*acrB and* Δ*tolC* strains. Values are averages of at least three independent experiments; error bars are sem (standard deviation/√n). Asterisks indicate *P*-values for the [AB*H] interaction term (see Methods). *P<0.05, **P<0.01; ***P<0.001, ns, not significant.

R was used for all statistical analyses of change in EOP (antibiotic effectiveness) or inducing concentration (dose) of ingredients [19]. For the antibiotic effectiveness experiments, we were interested in cases where the simultaneous exposure to both herbicide and antibiotic has a different effect on EOP than we would expect by looking at the effect of antibiotic and herbicide in isolation. These were tested on the log-transformed EOP scores using a multi-factor analysis of variance (ANOVA) and evaluating the significance of an (antibiotic*herbicide) interaction term (*P* values reported in Figs 1a, 2a and 3. See Table S1). Antibiotic concentrations were treated as separate categories. We tested for violations of assumptions of ANOVAs using residual plots. We fit these models using the lm function.

In the inducing concentration (dose) experiments, many data points were near or below the detection limit, and consequently the residuals from a standard ANOVA were not always normally distributed. In these cases, a Kruskal–Wallis one-way ANOVA, the equivalent nonparametric test, was used to test for differences in log-transformed EOP/EOP_0_ scores among herbicide concentrations (Figs 1b and 2b. See Table S1, available in the online version of this article). We present a *P* value for a comparison of a null model where log EOP/EOP_0_ is the same across all herbicide concentrations versus an alternative model where log EOP/EOP_0_ differs among some herbicide concentrations.

## Results

### Active ingredients of herbicides cause changes in antibiotic response

Responses to the antibiotics ampicillin (Amp), chloramphenicol (Cam), ciprofloxacin (Cip), kanamycin (Kan) and tetracycline (Tet) by *S. enterica* exposed to herbicide active ingredients were measured [15]. Bacteria were spread onto solid media with increasing concentrations of antibiotic, with either no or a single defined concentration of active ingredient (see Methods for details). The efficiency of plating (EOP) was determined for each defined concentration of antibiotic and active ingredient (Fig. 1a). The EOP is the ratio of the titre (c.f.u. ml^−1^) of a culture on treatment plates to the titre (c.f.u. ml^−1^) on LB. The limit of detection was determined by the number of bacteria in, and practical volumes of, culture that could be transferred to a Petri plate. This provided a range of 9 orders of magnitude (EOP ≈1-10^−8^).

The series of EOP measurements from high to low reveals the concentrations of antibiotic that affect survival and thus is indicative of antibiotic effectiveness. The active ingredient alone at the concentrations used did not reduce EOP. At low antibiotic concentrations the EOP was high, and it dropped below the detection limit as concentrations approached the MIC. In most cases the addition of active ingredient led to an increase in survival shown by higher EOP measurements at higher concentrations of antibiotic. However, decreases in survival and no change in response for some combinations of antibiotic and ingredient were also observed. The response to a single antibiotic could be different depending on the herbicide to which the bacteria were exposed. For example, glyphosate increased and 2,4-D decreased EOP on medium with Kan. Furthermore, the same herbicide could induce different responses depending on the antibiotic to which the bacteria were exposed. For example, 2,4-D increased EOP on Cam, but decreased it on Kan.

From the above we identified the antibiotic concentrations at which the EOP decreased by a factor of ≥10^3^ with or without herbicide exposure (Table 2). For the majority of combinations, herbicide exposure increased the antibiotic concentration 1.3–3.5 times. This was similar to what was observed for combinations of antibiotics and commercial formulations [15]. There were three combinations where the magnitude of change in antibiotic concentration was even larger. 2,4-D exposure decreased by four times the amount of Kan needed to cause a ≥10^3-^fold change in EOP, glyphosate increased the Kan concentration fivefold and dicamba exposure increased the Cam concentration sevenfold.

**Table 2.** Fold-change shift in antibiotic effectiveness following exposure to herbicide ingredients

	Amp	Cam	Cip	Kan	Tet
*S. enterica*					
Active ingredients					
Dicamba	1.3 (1500)	7 (1500)	3.5 (1500)	0 (1500)*	2.7 (1500)
2,4-D	ns	2.5 (600)	1.8 (5000)	4 (6000)*	2.2 (500)
Glyphosate	1.8 (3000)	1.5 (3000)*	2 (200)	5 (200)	1.4 (3000)*
Surfactants					
Tween80	ns	2.3	1.2	1.8	1.8
CMC	1.7	ns	ns	1.5	1.4
*E. coli*					
Surfactants					
Tween80	ns	1.6	0	1.5*	ns
CMC	1.25*	ns	ns	4	ns

Shown is fold-change in antibiotic concentration necessary to cause EOP to vary by a factor ≥1000 between treatment and no-treatment plates. The concentration of active ingredients used (in ppm ae) is given in parenthesis. Concentrations of Tween80 and CMC were 2 and 1 %, respectively. ns: not significant; 0: statistically significant differences, but the drop below the threshold of EOP 0.001 occurred at the same antibiotic concentration for treatment and no-treatment plates.

*Indicates a decrease in response.

At the antibiotic concentration that caused the biggest change in EOP (Fig. 1a), the minimum concentration, or dose, of ingredient that induced a response was determined (Fig. 1b). To aid visualization of the magnitude of change, we report the data as the log of the ratio of two EOP measurements: (1) EOP on antibiotic at a given active ingredient concentration to (2) EOP on antibiotic without active ingredient. Log-transformed EOP ratios >0 indicate that the bacteria survived higher concentrations of the antibiotic. Dicamba decreased (Kan) or increased (Amp, Cam, Cip, Tet) survival in the tested antibiotics by 10^3^–10^7^-fold (Table 3). The ratio for Tet did not reach statistical significance, but showed a clear trend. The pattern of responses was the same for both 2,4-D and dicamba, even though Cam did not reach statistical significance for the former, while Tet did. Using 2,4-D, EOP ratios reached 10^3^–10^5^. Glyphosate induced statistically significant decreases (Cam, Tet) or increases (Amp, Cip, Kan) in survival in all tested antibiotics, with EOP ratios reaching 10^4^–10^5^.

**Table 3.** Minimum concentration of ingredient required to cause a statistically significant (≥100-fold) change in EOP The maximum change in EOP ratios observed at any concentration is given in parenthesis. EOP ratios >1 indicate that bacteria become more tolerant to the antibiotic. Antibiotic concentrations, in µg ml^−1^ were: *S. enterica*: dicamba: 1.5/4.4/0.1/-/3.5. 2,4-D: -/4.4/0.05/6/2.5; glyphosate: 2.5/4/0.05/12/2.5; Tween80: -/4.4/0.03/12/2; CMC: 1.5/-/-/12/2.5; *E. coli*: Tween80: -/7.5/0.01/4/-; CMC: 5/-/-/10/- for Amp/Cam/Cip/Kan/Tet, respectively. nd: not determined because change in EOP was not significant. ns, not significant.

	Amp	Cam	Cip	Kan	Tet
*S. enterica*					
Active ingredients, ppm ae					
Dicamba	100 (8.7×10^3^)	1000 (1.2×10^7^)	1000 (3.7×10^7^)	nd	50 (1.5×10^5^)
2,4-D	nd	100 (3.3×10^5^)	7000 (8.9×10^5^)	6000 (1.3×10^−7^)	300 (3.1×10^3^)
Glyphosate	1500 (1.3×10^5^)	500 (7.3×10^−5^)	200 (2.8×10^5^)	125 (1.6×10^5^)	50 (1.2×10^−4^)
Surfactants %						
Tween80	nd	<0.05 (9.5×10^6^)	1 (4.1×10^4^)	2 (5.8×10^3^)	2 (1.3×10^6^)
CMC	ns (5.7×10^6^)	nd	nd	0.25 (4.8×10^5^)	1 (5.8×10^2^)
*E. coli*						
Surfactants, %						
Tween80	nd	1 (4.2×10^5^)	1.5 (1.2×10^3^)	0.5 (3.5×10^−5^)	nd
CMC	0.25 (3×10^−7^)	nd	nd	0.5 (1.8×10^5^)	nd

We restricted our comparisons to cases where there is a difference of at least 100-fold between the herbicide and non-herbicide treatments (Table 3). This criterion was in addition to statistical significance and makes our conclusions more conservative. A 100-fold threshold does not indicate that a lower value is biologically irrelevant, but represents a measure that our limit of detection could support. In most cases, the amount of active ingredient needed to meet the threshold was between 500 and 5000 ppm ae. Exceptions were glyphosate*Kan (125 ppm ae), *Cip (200 ppm ae) and *Tet (50 ppm ae). Kamba*Tet met the threshold at 50 ppm ae, but the effect was not statistically significant at this concentration.

### Common co-formulants Tween80 and CMC cause changes in antibiotic response

Commercial herbicide formulations contain a range of inert ingredients in addition to the herbicidal compounds [17, 18]. These include solvents, wetting agents and surfactants that can make up the non-water bulk of the formulation. Surfactants such as Tween80 are added to herbicide formulations to reduce the surface tension of water, which leads to a better distribution on leaf surfaces and aids uptake by the plant [20]. CMC is used as a binder or regulator of viscosity.

The change in EOP was measured at increasing concentrations of the different antibiotics on medium supplemented or not with co-formulants (Fig. 2a).For cost reasons we limited testing of active ingredients to only *S. enterica*. However, the low cost of surfactants allowed us to measure the responses of both *S. enterica* and *E. coli*.

Where a statistically significant change in EOP resulted from exposure to a co-formulant, the concentration of co-formulant needed to cause the response was then determined (Fig. 2b). As with the active ingredients, responses varied with compound, antibiotic and species. Exposure to some (9/20) combinations of co-formulant and antibiotic did not result in significant changes to EOP. Of the remaining (11/20) combinations, two decreased the concentration of antibiotic needed to meet our EOP threshold of a ≥10^3^-fold change in EOP (*E. coli* exposed to CMC*Amp and Tween80*Kan) and the other nine increased the necessary antibiotic concentrations. To quantify the effect, we identified the change in antibiotic concentration when the EOP changed by a factor of ≥10^3^ (Table 2). With the exception of CMC*Kan in *E. coli*, for which a fourfold increase in antibiotic concentration was observed, values were between 1.25- and 2.3-fold. Although often significant, the effects of co-formulants on antibiotic responses were generally weaker than those observed for the active ingredients.

The concentration (or dose) of co-formulant needed to induce a response is presented again as a log-transformed ratio of EOP measurements: (1) EOP on antibiotic at a given co-formulant concentration to (2) EOP on antibiotic without co-formulant. The *S. enterica* log EOP/EOP_0_ ratios were as high as 10^3^–10^6^ with exposure to Tween80, and 10^2^–10^6^ with exposure to CMC. The *E. coli* responses were mainly weaker than the *S. enterica* responses, but did reach 10^2^–10^7^ at their extremes (Table 3). Most of the combinations that induced a response caused a 100-fold change in EOP only towards the higher end of tested co-formulant concentrations (max. 2 % for Tween80 and 1 % for CMC) (Table 3). Exceptions were seen for *S. enterica* exposure to CMC*Kan (0.25 %) and Tween80*Cam (<0.05 %), and *E. coli* exposure to Tween80*Kan (0.5 %) and CMC*Amp (0.25 %).

We also tested the commercial wetting agent Pulse Penetrant which, according to the manufacturer, is used to ‘improve penetration and rainfastness of Roundup herbicide’, and contains organo-modified polydimethyl siloxane as the active ingredient. Pulse Penetrant did not induce significant changes to *S. enterica* antibiotic susceptibility for any of the five antibiotics when it was added at 2 %, 10 times the recommended application level. Any observed differences in EOP were <100-fold and only observed at one antibiotic concentration (data not shown).

### Efflux pumps are major contributors to increased resistance

Adaptive resistance can be caused by increases in efflux or decreases in influx [21]. This is achieved by regulating the expression of porins and efflux pumps. The efflux pump inhibitor PAβN (phenylalanine-arginine beta-naphthylamide) counteracted the response associated with exposure to herbicide formulations [15]. To investigate the role of several permeability and efflux components in resistance caused by the herbicides, five genes known to be involved in adaptive antibiotic resistance, four associated with efflux (*acrA*, *acrB*, *acrD*, *tolC*) and one associated with influx (*ompF*) were tested in a common genetic background called the Keio collection [22]. The Keio collection consists of a set of single gene deletion mutants of all non-essential genes of *E. coli* BW25113 (‘wild-type’).

Two antibiotics from different classes, Cip and Tet, and the commercial herbicide formulations Kamba and Roundup, were chosen to represent the response patterns observed previously. The change in EOP was measured at increasing concentrations of the different antibiotics on medium supplemented or not with herbicide.

The MIC of each herbicide and antibiotic was measured for each strain (Table S2). No change or an increase in the MIC of the herbicides was observed for the *∆ompF* and ∆*acrD* strains compared to the wild-type. A decrease in MIC was observed for strains with a ∆*acrA*, ∆*acrB,* or ∆*tolC* genotype. This effect was much more pronounced for Roundup than for Kamba. For these strains, the Roundup concentration used to measure changes in the EOP at different antibiotic concentrations was reduced from 1240 to 25 ppm ae, a concentration that caused no decrease in EOP by itself (Table 4, Fig. 3).

**Table 4.** Responses of gene deletion strains

	Kamba							Roundup						
	Cip				Tet			Cip				Tet		
Strain	*P*-value*	R^2^ (%)	Fold-change MIC	*P*-value*	R^2^ (%)	Fold-change MIC	*P*-value*	R^2^ (%)	Fold-change MIC	*P*-value*		R^2^ (%)	Fold-change MIC
BW25113 (WT)	***	6	3	***	4.9	2†	***	10.8	5	***		5.7	2
CR7000 (Δ*acrA)*	***	2.1	1.25	***	1.6	1.25	*	0.7	0	ns		0.3	0
CR5000 (Δ*acrB)*	ns	0.6	0	*	3	1.25†	***	1.8	0	***		1.2	0
JW5503 (Δ*tolC)*	***	6	2	***	0.8	0	ns	0.1	0	***		1.3	2
JW2454 (Δ*acrD)*	***	9.8	1.5†	***	8.1	2	***	7.9	2†	***		4.6	2.67
JW0912 (Δ*ompF)*	***	6.7	2.33	***	8.4	5	***	4.4	3.3	***		4.5	3†

*P-values for the interaction term. *P<0.05; **P<0.01; ***P<0.001. ns, not significant. See Materials and Methods for details.

†Indicates a decrease in response.

Fold-change MIC is change in the antibiotic concentration where EOP drops by at least a factor of 10^3^ compared to the no-herbicide treatment. Herbicide concentrations were 1380 ppm ae Kamba for all strains; 1240 ppm ae Roundup for WT, *ΔompF* and *ΔacrD*, and 25 ppm ae for Δ*acrA,* Δ*acrB* and Δ*tolC*.

R^2^ is a partial R^2^, describing the % of variability that is due to the antibiotic*herbicide interaction term.

Antibiotic susceptibility also varied because efflux has a role in adaptive resistance [21]. Consequently, a direct between-strain comparison of EOP at given antibiotic concentrations was not possible. Instead, the *P*-values for the antibiotic*herbicide interaction term were calculated for each genotype to determine whether the effects of the herbicides on antibiotic response could still be observed when the gene was deleted (Table 4). We also report the proportion of all variability in the dataset, which can be explained by the interaction term (partial R^2^). We express this measure as a percentage, with 0 % indicating that none of the variation in EOP is explained by the interaction term and 100 % indicating that all variability in the dataset is explained by the interaction term. Antibiotics and herbicide ingredients on their own explain the majority of variability in the dataset. The pattern observed for the wild-type resembled that seen for *E. coli* JB578 (the strain used in Ref [15]) and *S. enterica*. The addition of either herbicide led to an increase in survival on Cip, while Kamba increased and Roundup decreased survival on Tet. The results were statistically significant for all combinations, with the interaction explaining between 4.9 and 10.8 % of the variation in the wild-type (Table 4, Fig. 3. See Table S1). When the gene deletion strains were used, most combination exposures also produced highly significant interaction terms. This indicates that the deleted genes are not individually responsible for the entirety of the observed effects of the herbicides on adaptive resistance. However, for some combinations the interaction term was not significant. The Cip*Kamba interaction was disrupted for the *∆acrB* strain. For Cip*Roundup, deletions of *acrA* and *tolC* resulted in the disruption of the herbicide-induced effects. The Tet*Kamba interaction behaved similarly to Cip*Kamba. Only the *∆acrB* strain showed a weaker, albeit still significant, response to the Tet*Kamba exposure compared to the wild-type. The *∆acrA* strain did not respond to the combination of Tet*Roundup. The other strains did. This might hint at an interaction of *acrB* with other efflux pumps. For all herbicide*antibiotic combinations, R^2^ in the *acrA*, *∆acrB* and ∆*tolC* strains decreased relative to the wild-type, indicating that the genes may play a role in the observed effects.

Consistent with the observed role of efflux pumps, transcription of the *marRAB* operon increased when *E. coli* were exposed to the Tet*Kamba combination (Table S3). We chose this combination because resistance to Tet is mediated by the efflux pump TetA [23], and Kamba causes the same pattern of adaptive antibiotic resistance response as another benzoic acid derivative, salicylic acid, for which transcriptomes were previously investigated [24].

## Discussion

We show that bacteria exposed to the ingredients of herbicides respond differently to clinically relevant antibiotics. This is important because bacteria can be exposed to herbicides and antibiotics in environments as diverse as the human body, farms and in urban environments such as the lawns and gardens surrounding hospitals and homes. Exposure to herbicide active ingredients in food is regulated. Maximum residue levels (MRLs) of herbicides on food commodities are set by national and international bodies such as the Codex Alimentarius Commission (CAC) of the UN FAO. However, many other potential exposures in both urban and rural environments are not monitored. Thus, both the frequency of herbicide-induced changes in antibiotic resistance in any approved use of herbicides and antibiotics, and the effects of the change escape regulatory oversight.

Toxicity testing of herbicides before registration is usually restricted to the active ingredient tested in isolation, and in any case never extends to sub-lethal effects on potential human, companion animal or livestock pathogens. The concentrations of active ingredients at which change to antibiotic response was observed were within application levels, as would be seen in the field or urban environments [25]. They were higher than the MRL set by the CAC in food products. Several combinations produced effects at MRLs permissible in animal feed.

It is tempting to directly compare the results obtained for active ingredients with those for the respective commercial formulations. However, the active ingredients have different MICs and solubility compared to the commercial formulations, and hence the concentrations used often differed. For example, the glyphosate concentration in the commercial formulation used was 1240 ppm ae [15], while instead either 200 or 3000 ppm ae of the pure active ingredient was used here. However, in all cases the responses caused by commercial formulations and the corresponding active ingredients were in the same direction, only the magnitude of the effects varying. The responses elicited by the tested co-formulants, albeit generally weaker than those of the active ingredients, suggest that effects might be modulated by co-formulants present in the formulations.

In commercial use, active ingredients are mixed with co-formulants to improve spreading, absorbing or penetrating properties. Toxicity data for these co-formulants are not usually required by regulators, and residues of co-formulants left by herbicides are not monitored. The concentrations of Tween80 and CMC at which change to antibiotic response was observed were within levels permitted in food. The United States Food and Drug Administration guidelines allow concentrations of Tween80 up to 1 % in some processed foods, where it is used as an emulsifier. Herbicide formulations before dilution are exempt from this concentration limit [26]. Herbicide formulations contain binders such as CMC in the range of 2–10 % by weight [27, 28]. CMC has ‘generally regarded as safe’ status and can be found in food at concentrations up to 2 % [29].

It is noteworthy that the tested co-formulants did not induce the same pattern of antibiotic response observed for any of the herbicide formulations. While we observed mainly decreases in antibiotic susceptibility or no effect induced by the two co-formulants and a commercial wetting agent, increases in susceptibility to some of these co-formulants have been reported elsewhere. For example, susceptibility of *Streptococcus agalactiae* to platensimycin and triclosan was increased by as little as 0.2 % Tween80, while susceptibility to triclosan increased and to platensimycin decreased for MRSA when Tween80 was added [30].

Adaptive responses leading to antibiotic cross-response have been reported in many bacterial species upon sub-lethal exposure to a variety of substances, including bile salts [31], weak acids [12], triclosan, chlorhexidine [32], quaternary ammonium compounds [33], and other antibiotics [8, 34]. We add to the list herbicides and some inert ingredients of commercial products. The relevance of this work extends beyond herbicides because other commercial products use the same co-formulants. For example, a patent for fluoroquinolone compositions specified using various polysorbates, including Tween80, as surfactants, co-solvents, or emulsifiers in concentrations of up to 6.5 % by weight [35]. We found increased survival of both *E. coli* and *S. enterica* in higher concentrations of the fluoroquinolone antibiotic Cip at exposures of only 2 % Tween80. It is worth investigating whether the choice of co-formulants will influence the frequency of resistance or the efficacy of treatments using these drugs. In addition to use in processed foods, polysorbates are widely used as solubilising agents in products intended for applications that result in chronic human exposures. These products include cosmetics, mouthwash, and medical preparations. Thus, the patchwork of non-antibiotic resistance-inducing chemicals of human origin that microbes routinely encounter may conflict with antibiotic stewardship strategies, undermining goals to substantially reduce antibiotic resistance, or sustain susceptibility, even if antibiotic use were decreased.

The phenotype and transcription assays confirmed that multiple genes respond to herbicide exposures, and some also contributed to adaptive resistance. Several genes known to participate in adaptive resistance were tested in phenotype assays and by transcriptomics. Three strains with gene deletions had a different phenotypic pattern of response compared to the wild-type. These had deletions in ∆*acrA* (Roundup*Tet), ∆*acrB* (Kamba*Cip), or ∆*tolC* (Roundup*Cip). For these combinations, the deleted gene explained most if not all of the adaptive response. No other statistically robust interactions were observed. It is noteworthy that the herbicide concentrations tolerated by these strains were much lower than those tolerated by the wild-type, confirming that adaptive resistance contributes to the intrinsic herbicide and antibiotic response level. This is consistent with an increase in *acrB* (efflux) and a decrease in *ompF* (influx) transcripts observed for Tet*Kamba interactions. In contrast, AcrD did not contribute to the response to either antibiotic or Kamba. Because deleting some components of the AcrAB-TolC system did not uniformly impact responses to herbicide/antibiotic combinations, the response of *E. coli* to the compounds tested probably involves additional genes [36]. This suggestion is consistent with a *mar*-dependent and an undescribed *mar*-independent pathway as suggested by others [37]. The data presented here indicate that genes outside the RND family of transporters contribute to herbicide-induced adaptive antibiotic resistance. Our survey has not been exhaustive, but points at a complex response by bacteria exposed to herbicides and provides the basis for further work identifying herbicide-responsive gene networks in bacteria.

Multiple strategies are needed to address the antibiotic resistance crisis. While new drugs will certainly be required, invention alone will fail unless we can maintain the effectiveness of antimicrobial agents, both old and new [38]. Sustainable use of antibiotics will likely include cycling drugs out of use for periods of time. This approach may be compromised if other environmental factors sustain resistance – possibly even favouring the development of populations with acquired resistance – or select for rapid adaptation of bacteria when the drug is re-introduced. It will only be possible to avoid rapid evolution of resistance to new drugs, or engineer a return to susceptibility to existing drugs, if we understand the complex interaction of microbes with multiple chemical releases into the environment.

Antibiotic resistance is influenced by more factors than just exposure of bacteria to antibiotics. These other factors include manufactured products released into the environment. We demonstrated that the pure forms of dicamba, 2,4-D, and glyphosate, as well as common surfactants, can change the susceptibility of bacteria to a diverse range of antibiotics upon concurrent exposure. The inducing concentrations were well within the working concentrations of herbicides to which people, pets and farm animals may be exposed.

## Supplementary Data

455 Supplementary File 2

## References

[1] de Kraker ME, Davey PG, Grundmann H, Burden study group (2011). Mortality and hospital stay associated with resistant *Staphylococcus aureus* and *Escherichia coli* bacteremia: estimating the burden of antibiotic resistance in Europe. PLoS Med.

[2] Lemos EV, de La Hoz FP, Einarson TR, McQhan WF, Quevedo E (2014). Carbapenem resistance and mortality in patients with *Acinetobacter baumannii* infection: systematic review and meta-analysis. Clin Microbiol Infect.

[3] Robinson TP, Bu DP, Carrique-Mas J, Fèvre EM, Gilbert M (2016). Antibiotic resistance is the quintessential one health issue. Trans R Soc Trop Med Hyg.

[4] Andersson DI, Hughes D (2014). Microbiological effects of sublethal levels of antibiotics. Nat Rev Microbiol.

[5] Falagas ME, Tansarli GS, Rafailidis PI, Kapaskelis A, Vardakas KZ (2012). Impact of antibiotic MIC on infection outcome in patients with susceptible Gram-negative bacteria: a systematic review and meta-analysis. Antimicrob Agents Chemother.

[6] Willms AR, Roughan PD, Heinemann JA (2006). Static recipient cells as reservoirs of antibiotic resistance during antibiotic therapy. Theor Popul Biol.

[7] Heinemann JA (1999). How antibiotics cause antibiotic resistance. Drug Discov Today.

[8] Fernández L, Breidenstein EB, Hancock RE (2011). Creeping baselines and adaptive resistance to antibiotics. Drug Resist Updat.

[9] Elkins CA, Mullis LB (2007). Substrate competition studies using whole-cell accumulation assays with the major tripartite multidrug efflux pumps of *Escherichia coli*. Antimicrob Agents Chemother.

[10] Guo X, Murray M, Xiong C, Zhu S (2013). Treatment of *Eschericia coli* BW25113 with subinhibitory levels of kanamycin results in antibiotic cross-resistance and TolC upregulation. J Exp Microbiol Immun.

[11] Price CT, Lee IR, Gustafson JE (2000). The effects of salicylate on bacteria. Int J Biochem Cell Biol.

[12] Rosner JL (1985). Nonheritable resistance to chloramphenicol and other antibiotics induced by salicylates and other chemotactic repellents in *Escherichia coli* K-12. Proc Natl Acad Sci USA.

[13] Prouty AM, Brodsky IE, Falkow S, Gunn JS (2004). Bile-salt-mediated induction of antimicrobial and bile resistance in Salmonella typhimurium. Microbiology.

[14] Ferran AA, Bibbal D, Pellet T, Laurentie M, Gicquel-Bruneau M (2013). Pharmacokinetic/pharmacodynamic assessment of the effects of parenteral administration of a fluoroquinolone on the intestinal microbiota: comparison of bactericidal activity at the gut versus the systemic level in a pig model. Int J Antimicrob Agents.

[15] Kurenbach B, Marjoshi D, Amábile-Cuevas CF, Ferguson GC, Godsoe W (2015). Sublethal exposure to commercial formulations of the herbicides dicamba, 2,4-dichlorophenoxyacetic acid, and glyphosate cause changes in antibiotic susceptibility in *Escherichia coli* and *Salmonella enterica* serovar Typhimurium. MBio.

[16] EPA Inert Ingredients Regulation. www.epa.gov/pesticide-registration/inert-ingredients-regulation.

[17] Arnold KA (1994). Improved glyphosate herbicide formulation patent EP 0582561 A1.

[18] Mito N (2000). Synergistic herbicidal compositions comprising glyphosate patent CA 2374163 A1.

[19] (2013). R: a language and environment for statistical computing [database on the Internet]. R Foundation for Statistical Computing. http://R-project.org.

[20] Estrine B, Marinkovic S, Kuenemann P, Lajoie C, Paris A (2014). Herbicide composition having improved effectiveness, method of preparation and use patent US 8709978 B2.

[21] Fernández L, Hancock RE (2012). Adaptive and mutational resistance: role of porins and efflux pumps in drug resistance. Clin Microbiol Rev.

[22] Baba T, Ara T, Hasegawa M, Takai Y, Okumura Y (2006). Construction of *Escherichia coli* K-12 in-frame, single-gene knockout mutants: the Keio collection. Mol Syst Biol.

[23] Hillen W, Berens C (1994). Mechanisms underlying expression of Tn10 encoded tetracycline resistance. Annu Rev Microbiol.

[24] Pomposiello PJ, Bennik MH, Demple B (2001). Genome-wide transcriptional profiling of the *Escherichia coli* responses to superoxide stress and sodium salicylate. J Bacteriol.

[25] CAC (2014). Report of the 46th Session of the Codex Committee on Pesticide Residues.

[26] FDA (2016). Code of Federal Regulations 21 CFR I B 172.1.172.840 (12).

[27] Grundman O, Khozin-Goldberg I, Hacohen Z, Shapira M, Raveh D (2016). Compositions and methods for conferring herbicide resistance patent US 9267129 B2.

[28] Ushiguchi Y, Hori Y, Takahashi K, Yamamoto S I (2006). Granular herbicide patent US 7094734 B2.

[29] Chassaing B, Koren O, Goodrich JK, Poole AC, Srinivasan S (2015). Dietary emulsifiers impact the mouse gut microbiota promoting colitis and metabolic syndrome. Nature.

[30] Krsta D, Ku C, Crosby IT, Capuano B, Manallack DT (2014). Bacterial fatty acid synthesis: effects of tween 80 on antibiotic potency against *Streptococcus agalactiae* and methicillin-resistant *Staphylococcus aureus*. Anti-Infective Agents.

[31] Noriega L, de Los Reyes-Gavilán CG, Margolles A (2005). Acquisition of bile salt resistance promotes antibiotic susceptibility changes in bifidobacterium. J Food Prot.

[32] Braoudaki M, Hilton AC (2004). Low level of cross-resistance between triclosan and antibiotics in *Escherichia coli* K-12 and *E. coli* O55 compared to *E. coli* O157. FEMS Microbiol Lett.

[33] Buffet-Bataillon S, Tattevin P, Maillard JY, Bonnaure-Mallet M, Jolivet-Gougeon A (2016). Efflux pump induction by quaternary ammonium compounds and fluoroquinolone resistance in bacteria. Future Microbiol.

[34] Lázár V, Nagy I, Spohn R, Csörgő B, Györkei Á (2014). Genome-wide analysis captures the determinants of the antibiotic cross-resistance interaction network. Nat Commun.

[35] Tyle P, Gupta PK, Norton SE, Brunner L, Blondeau J (2015). Bausch & Lomb Inc., Rochester, USA, assignee. Compositions and methods for treating, reducing, ameliorating, or preventing infections caused by antibacterial drug-resistant bacteria patent US 8937062 B2.

[36] Tal N, Schuldiner S (2009). A coordinated network of transporters with overlapping specificities provides a robust survival strategy. Proc Natl Acad Sci USA.

[37] Cohen SP, Levy SB, Foulds J, Rosner JL (1993). Salicylate induction of antibiotic resistance in *Escherichia coli*: activation of the mar operon and a mar-independent pathway. J Bacteriol.

[38] Amábile-Cuevas C (2016). Society must seize control of the antibiotics crisis. Nature.

[39] Ruiz C, Levy SB (2014). Regulation of acrAB expression by cellular metabolites in *Escherichia coli*. J Antimicrob Chemother.

[40] Roantree RJ, Kuo TT, MacPhee DG (1977). The effect of defined lipopolysaccharide core defects upon antibiotic resistances of *Salmonella typhimurium*. J Gen Microbiol.

