# Supplementary material for: Herbicide ingredients change Salmonella enterica sv. Typhimurium and Escherichia coli antibiotic responses
Source: Microbiology (Reading). 2017 Nov 17;163(12):1791–801. doi: 10.1099/mic.0.000573 (PMC5845734; doi:10.1099/mic.0.000573)
Supplement: Supplementary File 2 [file mic-163-1791-s001.pdf]

Supplementary Material

Herbicide ingredients change *Salmonella enterica* sv. Typhimurium and *Escherichia coli* antibiotic tolerances

Brigitta Kurenbach, Paddy S. Gibson, Amy M. Hill, Adam S. Bitzer, Mark W. Silby, William Godsoe, and Jack A. Heinemann

Supplementary Table 1: ANOVA tables for data in Figures 1-3

Figure 1a – *S. enterica*

Dicamba

Amp

	Df	Sum Sq	Mean Sq	F value	Pr(>F)	
H	1	93.1	93.14	17.094	0.000514	***
AB	4	1069.2	267.31	49.058	4.62e-10	***
H:AB	4	115.6	28.91	5.305	0.004444	**
Residuals	20	109.0	5.45			

Cam

	Df	Sum Sq	Mean Sq	F value	Pr(>F)	
H	1	570.4	570.4	274.55	3.79e-13	***
AB	4	765.0	191.3	92.05	1.38e-12	***
H:AB	4	281.7	70.4	33.89	1.20e-08	***
Residuals	20	41.6	2.1			

Cip

	Df	Sum Sq	Mean Sq	F value	Pr(>F)	
H	1	324.7	324.7	121.11	6.18e-10	***
AB	4	982.3	245.6	91.60	1.45e-12	***
H:AB	4	324.6	81.1	30.27	3.14e-08	***
Residuals	20	53.6	2.7			

Kan

	Df	Sum Sq	Mean Sq	F value	Pr(>F)	
H	1	12.9	12.95	6.348	0.02037	*
AB	4	1122.1	280.52	137.525	3.01e-14	***
H:AB	4	48.4	12.09	5.929	0.00258	**
Residuals	20	40.8	2.04			

Tet

	Df	Sum Sq	Mean Sq	F value	Pr(>F)	
H	1	646.9	646.9	52.018	1.17e-07	***
AB	6	689.3	114.9	9.238	1.87e-05	***
H:AB	6	238.2	39.7	3.192	0.0175	*
Residuals	26	323.3	12.4			

2,4-D

Amp

	Df	Sum Sq	Mean Sq	F value	Pr(>F)	
H	1	100.6	100.63	7.813	0.0112	*
AB	4	992.3	248.07	19.260	1.24e-06	***
H:AB	4	100.5	25.11	1.950	0.1414	
Residuals	20	257.6	12.88			

Cam

	Df	Sum Sq	Mean Sq	F value	Pr(>F)	
H	1	344.0	344.0	412.16	8.11e-15	***
AB	4	1048.8	262.2	314.17	< 2e-16	***
H:AB	4	324.6	81.2	97.25	8.22e-13	***
Residuals	20	16.7	0.8			

Cip

	Df	Sum Sq	Mean Sq	F value	Pr(>F)	
H	1	145.0	144.99	118.43	7.49e-10	***
AB	4	932.4	233.10	190.40	1.29e-15	***
H:AB	4	187.8	46.94	38.34	4.11e-09	***
Residuals	20	24.5	1.22			

Kan

	Df	Sum Sq	Mean Sq	F value	Pr(>F)	
H	1	464.8	464.8	117.724	7.89e-10	***
AB	4	800.2	200.0	50.664	3.45e-10	***
H:AB	4	147.6	36.9	9.348	0.000199	***
Residuals	20	79.0	3.9			

Tet

	Df	Sum Sq	Mean Sq	F value	Pr(>F)	
H	1	93.2	93.24	40.14	1.50e-06	***
AB	5	1346.2	269.24	115.90	5.37e-16	***
H:AB	5	69.6	13.92	5.99	0.000986	***
Residuals	24	55.8	2.32			

Glyphosate

Amp

	Df	Sum Sq	Mean Sq	F value	Pr(>F)	
H	1	207.8	207.77	47.29	1.11e-06	***
AB	4	892.1	223.03	50.77	3.39e-10	***
H:AB	4	251.5	62.88	14.31	1.14e-05	***
Residuals	20	87.9	4.39			

Cam

	Df	Sum Sq	Mean Sq	F value	Pr(>F)	
H	1	174.7	174.71	14.966	0.000956	***
AB	4	1156.0	289.01	24.757	1.67e-07	***
H:AB	4	190.4	47.61	4.078	0.014107	*
Residuals	20	233.5	11.67			

Cip

	Df	Sum Sq	Mean Sq	F value	Pr(>F)	
H	1	63.6	63.6	47.99	1.00e-06	***
AB	4	1305.1	326.3	246.08	< 2e-16	***
H:AB	4	75.1	18.8	14.16	1.23e-05	***
Residuals	20	26.5	1.3			

Kan

	Df	Sum Sq	Mean Sq	F value	Pr(>F)	
H	1	345.4	345.4	405.04	8.69e-13	***
AB	3	659.9	220.0	257.95	9.38e-14	***
H:AB	3	222.2	74.1	86.85	4.08e-10	***
Residuals	16	13.6	0.9			

Tet

	Df	Sum Sq	Mean Sq	F value	Pr(>F)	
H	1	185.8	185.80	36.341	6.81e-06	***
AB	4	872.6	218.15	42.669	1.60e-09	***
H:AB	4	137.9	34.48	6.743	0.00132	**
Residuals	20	102.3	5.11			

Figure 1b – *S. enterica*

Dicamba

Amp

	Df	Sum Sq	Mean Sq	F value	Pr(>F)	
T	4	167.28	41.82	6.281	0.00856	**
Residuals	10	66.58	6.66			

Cam

	Df	Sum Sq	Mean Sq	F value	Pr(>F)	
T	4	632.3	158.1	16.12	0.000232	***
Residuals	10	98.0	9.8			

Cip

Kruskal-Wallis chi-squared = 9.5957, df = 3, p-value = 0.02233 *

Kan

Kruskal-Wallis chi-squared = 12.624, df = 4, p-value = 0.01327 *

Tet

Kruskal-Wallis chi-squared = 5.036, df = 4, p-value = 0.2836

2,4-D

Cam

Kruskal-Wallis chi-squared = 10.846, df = 5, p-value = 0.05452

Cip

	Df	Sum Sq	Mean Sq	F value	Pr(>F)	
T	4	242.9	60.73	4.106	0.0319	*
Residuals	10	147.9	14.79			

Kan

	Df	Sum Sq	Mean Sq	F value	Pr(>F)	
T	3	106.62	35.54	43.02	2.79e-05	***
Residuals	8	6.61	0.83			

Tet

Kruskal-Wallis chi-squared = 12.523, df = 4, p-value = 0.01386 *

Glyphosate

Amp

	Df	Sum Sq	Mean Sq	F value	Pr(>F)	
T	5	935.1	187.01	66.63	2.47e-08	***
Residuals	12	33.7	2.81			

Cam

	Df	Sum Sq	Mean Sq	F value	Pr(>F)	
T	3	305.17	101.72	22.91	0.000278	***
Residuals	8	35.52	4.44			

Cip

Kruskal-Wallis chi-squared = 11.415, df = 4, p-value = 0.02228 *

Kan

	Df	Sum Sq	Mean Sq	F value	Pr(>F)	
T	4	415.2	103.8	11.93	0.000803	***
Residuals	10	87.0	8.7			

Tet

	Df	Sum Sq	Mean Sq	F value	Pr(>F)	
T	4	178.90	44.73	10.52	0.00132	**
Residuals	10	42.52	4.25			

Figure 2a

Tween80 – *S. enterica*

Amp

	Df	Sum Sq	Mean Sq	F value	Pr(>F)	
H	1	2.8	2.8	2.508	0.126	
AB	5	1801.9	360.4	318.422	<2e-16	***
H:AB	5	4.4	0.9	0.769	0.581	
Residuals	24	27.2	1.1			

Cam

	Df	Sum Sq	Mean Sq	F value	Pr(>F)	
H	1	414.8	414.8	325.93	1.80e-15	***
AB	5	1248.7	249.7	196.24	< 2e-16	***
H:AB	5	283.6	56.7	44.58	2.24e-11	***
Residuals	24	30.5	1.3			

Cip

	Df	Sum Sq	Mean Sq	F value	Pr(>F)	
H	1	118.4	118.4	50.46	2.42e-07	***
AB	5	1683.6	336.7	143.54	< 2e-16	***
H:AB	5	163.8	32.8	13.97	1.90e-06	***
Residuals	24	56.3	2.3			

Kan

	Df	Sum Sq	Mean Sq	F value	Pr(>F)	
H	1	39.9	39.93	12.582	0.00164	**
AB	5	1167.5	233.51	73.573	9.26e-14	***
H:AB	5	78.7	15.73	4.957	0.00294	**
Residuals	24	76.2	3.17			

Tet

	Df	Sum Sq	Mean Sq	F value	Pr(>F)	
H	1	145.0	144.97	43.45	8.11e-07	***
AB	5	1458.0	291.61	87.39	1.34e-14	***
H:AB	5	196.0	39.21	11.75	8.05e-06	***
Residuals	24	80.1	3.34			

Tween80 – *E. coli*

Amp

	Df	Sum Sq	Mean Sq	F value	Pr(>F)	
H	1	3.61	3.61	0.479	0.49566	
AB	5	311.21	62.24	8.244	0.00012	***
H:AB	5	112.14	22.43	2.970	0.03167	*
Residuals	24	181.20	7.55			

Cam

	Df	Sum Sq	Mean Sq	F value	Pr(>F)	
H	1	187.2	187.21	34.561	9.46e-06	***
AB	4	732.2	183.04	33.793	1.23e-08	***
H:AB	4	89.8	22.44	4.143	0.0132	*
Residuals	20	108.3	5.42			

Cip

	Df	Sum Sq	Mean Sq	F value	Pr(>F)	
H	1	27.8	27.8	68.92	6.55e-08	***
AB	4	1500.5	375.1	930.00	< 2e-16	***
H:AB	4	27.3	6.8	16.91	3.34e-06	***
Residuals	20	8.1	0.4			

Kan

	Df	Sum Sq	Mean Sq	F value	Pr(>F)	
H	1	18.5	18.54	60.65	1.76e-07	***
AB	4	778.5	194.63	636.82	< 2e-16	***
H:AB	4	57.9	14.47	47.34	6.36e-10	***
Residuals	20	6.1	0.31			

Tet

	Df	Sum Sq	Mean Sq	F value	Pr(>F)	
H	1	2.6	2.62	0.842	0.368	
AB	5	1104.1	220.81	71.022	1.37e-13	***
H:AB	5	5.4	1.07	0.345	0.881	
Residuals	24	74.6	3.11			

CMC – *S. enterica*

Amp

	Df	Sum Sq	Mean Sq	F value	Pr(>F)	
H	1	34.4	34.4	3.584	0.0729	.
AB	4	1626.9	406.7	42.374	1.71e-09	***
H:AB	4	114.7	28.7	2.987	0.0438	*
Residuals	20	192.0	9.6			

Cam

	Df	Sum Sq	Mean Sq	F value	Pr(>F)	
H	1	0.0	0.0	0.006	0.936	
AB	4	1829.7	457.4	142.754	2.32e-16	***
H:AB	4	0.1	0.0	0.007	1.000	
Residuals	24	76.9	3.2			

Cip

	Df	Sum Sq	Mean Sq	F value	Pr(>F)	
H	1	0.7	0.7	0.821	0.376	
AB	4	1706.3	426.6	531.849	<2e-16	***
H:AB	4	1.5	0.4	0.471	0.757	
Residuals	20	16.0	0.8			

Kan

	Df	Sum Sq	Mean Sq	F value	Pr(>F)	
H	1	170.2	170.16	229.51	2.00e-12	***
AB	4	682.6	170.65	230.17	< 2e-16	***
H:AB	4	158.6	39.65	53.48	2.12e-10	***
Residuals	20	14.8	0.74			

Tet

	Df	Sum Sq	Mean Sq	F value	Pr(>F)	
H	1	101.4	101.38	28.620	3.09e-05	***
AB	4	999.6	249.91	70.551	1.67e-11	***
H:AB	4	69.8	17.46	4.929	0.00626	**
Residuals	20	70.8	3.54			

CMC – *E. coli*

Amp

	Df	Sum Sq	Mean Sq	F value	Pr(>F)	
H	1	178.4	178.45	228.57	5.36e-15	***
AB	6	939.9	156.65	200.66	< 2e-16	***
H:AB	6	107.9	17.98	23.02	1.27e-09	***
Residuals	28	21.9	0.78			

Cam

	Df	Sum Sq	Mean Sq	F value	Pr(>F)	
H	1	1.8	1.84	0.645	0.430	
AB	5	1461.5	292.30	102.513	2.18e-15	***
H:AB	5	2.2	0.44	0.155	0.976	
Residuals	24	68.4	2.85			

Cip

	Df	Sum Sq	Mean Sq	F value	Pr(>F)	
H	1	1.8	1.8	1.552	0.225	
AB	5	1644.1	328.8	281.303	<2e-16	***
H:AB	5	5.3	1.1	0.898	0.498	
Residuals	24	28.1	1.2			

Kan

	Df	Sum Sq	Mean Sq	F value	Pr(>F)	
H	1	243.0	243.00	81.717	8.51e-10	***
AB	6	736.5	122.75	41.281	1.22e-12	***
H:AB	6	153.5	25.58	8.601	2.40e-05	***
Residuals	28	83.3	2.97			

Tet

	Df	Sum Sq	Mean Sq	F value	Pr(>F)	
H	1	48.8	48.83	9.011	0.00485	**
AB	8	1589.9	198.74	36.673	4.75e-15	***
H:AB	8	63.7	7.96	1.469	0.20292	
Residuals	36	195.1	5.42			

Figure 2b

<u>Tween80 – *S. enterica*</u>

Cam

	Df	Sum Sq	Mean Sq	F value	Pr(>F)	
T	8	556.3	69.54	5.209	0.00177	**
Residuals	18	240.3	13.35			

Cip

	Df	Sum Sq	Mean Sq	F value	Pr(>F)	
T	7	616.9	88.13	24.24	2.21e-07	***
Residuals	16	58.2	3.64			

Kan

	Df	Sum Sq	Mean Sq	F value	Pr(>F)	
T	6	134.84	22.47	3.888	0.017	*
Residuals	14	80.92	5.78			

Tet

	Df	Sum Sq	Mean Sq	F value	Pr(>F)	
T	6	196.78	32.80	9.271	0.000325	***
Residuals	14	49.52	3.54			

<u>CMC – *S. enterica*</u>

Amp

Kruskal-Wallis chi-squared = 2.1823, df = 4,
p-value = 0.7023

Kan

	Df	Sum Sq	Mean Sq	F value	Pr(>F)	
T	4	431.9	107.97	107	3.65e-08	***
Residuals	10	10.1	1.01			

Tet

Kruskal-Wallis chi-squared = 13.04, df = 4,
p-value = 0.01108 *

<u>Tween80 – *E. coli*</u>

Cam

	Df	Sum Sq	Mean Sq	F value	Pr(>F)	
T	7	476.1	68.02	47.6	1.55e-09	***
Residuals	16	22.9	1.43			

Cip

	Df	Sum Sq	Mean Sq	F value	Pr(>F)	
T	7	163.98	23.426	19.77	9.27e-07	***
Residuals	16	18.95	1.185			

Kan

	Df	Sum Sq	Mean Sq	F value	Pr(>F)	
T	7	296.96	42.42	763.6	<2e-16	***
Residuals	16	0.89	0.06			

<u>CMC – *E. coli*</u>

Amp

Kruskal-Wallis chi-squared = 12.926, df = 4,
p-value = 0.01164 *

Kan

	Df	Sum Sq	Mean Sq	F value	Pr(>F)	
T	4	380.8	95.19	63.77	4.44e-07	***
Residuals	10	14.9	1.49			

Figure 3 – KEIO knock out strains

BW25113 (WT) – Cip – Kamba

	Df	Sum Sq	Mean Sq	F value	Pr(>F)	
H	1	39.1	39.09	175.71	3.19e-16	***
AB	9	1867.7	207.52	932.86	< 2e-16	***
H:AB	9	141.7	15.74	70.76	< 2e-16	***
Residuals	40	8.9	0.22			

BW25113 (WT) – Cip – Roundup

	Df	Sum Sq	Mean Sq	F value	Pr(>F)	
H	1	177.5	177.47	3461.0	<2e-16	***
AB	9	1832.7	203.63	3971.2	<2e-16	***
H:AB	9	414.8	46.09	898.9	<2e-16	***
Residuals	40	2.1	0.05			

BW25113 (WT) – Tet – Kamba

	Df	Sum Sq	Mean Sq	F value	Pr(>F)	
H	1	569.2	569.2	4204.7	<2e-16	***
AB	9	1867.6	207.5	1532.8	<2e-16	***
H:AB	9	361.8	40.2	296.9	<2e-16	***
Residuals	40	5.4	0.1			

BW25113 (WT) – Tet – Roundup

	Df	Sum Sq	Mean Sq	F value	Pr(>F)	
H	1	31.8	31.79	100.50	1.8e-12	***
AB	9	1919.2	213.24	674.09	< 2e-16	***
H:AB	9	119.8	13.32	42.09	< 2e-16	***
Residuals	40	12.7	0.32			

JW0912 (Δ*ompF*) – Cip – Kamba

	Df	Sum Sq	Mean Sq	F value	Pr(>F)	
H	1	123	122.57	142.3	<2e-16	***
AB	12	3399	283.25	328.7	<2e-16	***
H:AB	12	256	21.37	24.8	<2e-16	***
Residuals	52	45	0.86			

JW0912 (Δ*ompF*) – Cip – Roundup

	Df	Sum Sq	Mean Sq	F value	Pr(>F)	
H	1	937.3	937.3	628.20	<2e-16	***
AB	12	2369.7	197.5	132.36	<2e-16	***
H:AB	12	627.0	52.2	35.02	<2e-16	***
Residuals	52	77.6	1.5			

JW0912 (Δ*ompF*) – Tet – Kamba

	Df	Sum Sq	Mean Sq	F value	Pr(>F)	
H	1	295.4	295.41	89.99	6.22e-13	***
AB	12	2913.3	242.78	73.96	< 2e-16	***
H:AB	12	592.4	49.37	15.04	6.23e-13	***
Residuals	52	170.7	3.28			

JW0912 (Δ*ompF*) – Tet – Roundup

	Df	Sum Sq	Mean Sq	F value	Pr(>F)	
H	1	14.3	14.26	27.53	2.89e-06	***
AB	12	3028.2	252.35	487.03	< 2e-16	***
H:AB	12	150.1	12.51	24.14	< 2e-16	***
Residuals	52	26.9	0.52			

JW2454 (Δ*acr*D) - Cip - Kamba

	Df	Sum Sq	Mean Sq	F value	Pr(>F)	
H	1	75.0	75.02	18.622	0.000143	***
AB	7	2046.1	292.30	72.559	< 2e-16	***
H:AB	7	281.5	40.21	9.982	1.49e-06	***
Residuals	32	128.9	4.03			

JW2454 (Δ*acr*D) - Cip - Roundup

	Df	Sum Sq	Mean Sq	F value	Pr(>F)	
H	1	336.3	336.3	139.02	3.48e-13	***
AB	7	1645.3	235.0	97.17	< 2e-16	***
H:AB	7	345.1	49.3	20.38	4.01e-10	***
Residuals	32	77.4	2.4			

JW2454 (Δ*acr*D) - Tet - Kamba

	Df	Sum Sq	Mean Sq	F value	Pr(>F)	
H	1	132.5	132.46	407.82	<2e-16	***
AB	8	1753.4	219.18	674.80	<2e-16	***
H:AB	8	247.8	30.98	95.38	<2e-16	***
Residuals	36	11.7	0.32			

JW2454 (Δ*acr*D) - Tet - Roundup

	Df	Sum Sq	Mean Sq	F value	Pr(>F)	
H	1	373.1	373.1	378.27	< 2e-16	***
AB	8	2054.7	256.8	260.39	< 2e-16	***
H:AB	8	243.2	30.4	30.82	7.07e-14	***
Residuals	36	35.5	1.0			

CR7000 (ΔacrA) - Cip - Kamba

	Df	Sum Sq	Mean Sq	F value	Pr(>F)	
H	1	47	46.8	172.91	<2e-16	***
AB	13	4179	321.5	1188.75	<2e-16	***
H:AB	13	93	7.2	26.54	<2e-16	***
Residuals	56	15	0.3			

CR7000 (Δ*acr*A) - Cip - Roundup

	Df	Sum Sq	Mean Sq	F value	Pr(>F)	
H	1	1	0.7	0.627	0.4316	
AB	13	4249	326.9	287.213	<2e-16	***
H:AB	13	29	2.2	1.973	0.0407	*
Residuals	56	64	1.1			

CR7000 (Δ*acr*A) - Tet - Kamba

	Df	Sum Sq	Mean Sq	F value	Pr(>F)	
H	1	24.3	24.35	42.967	7.85e-08	***
AB	9	2141.2	237.91	419.849	< 2e-16	***
H:AB	9	34.7	3.86	6.804	7.36e-06	***
Residuals	40	22.7	0.57			

CR7000 (Δ*acr*A) - Tet - Roundup

	Df	Sum Sq	Mean Sq	F value	Pr(>F)	
H	1	2.3	2.30	2.165	0.149	
AB	9	2491.0	276.78	260.835	<2e-16	***
H:AB	9	8.6	0.96	0.900	0.534	
Residuals	40	42.4	1.06			

CR5000 (Δ*acr*B) - Cip - Kamba

	Df	Sum Sq	Mean Sq	F value	Pr(>F)	
H	1	4.9	4.92	10.420	0.00317	**
AB	6	1009.8	168.30	356.567	< 2e-16	***
H:AB	6	5.9	0.98	2.078	0.08804	.
Residuals	28	13.2	0.47			

CR5000 (Δ*acr*B) - Cip - Roundup

	Df	Sum Sq	Mean Sq	F value	Pr(>F)	
H	1	12.4	12.38	37.17	1.41e-06	***
AB	6	1136.3	189.38	568.47	< 2e-16	***
H:AB	6	22.2	3.69	11.09	2.51e-06	***
Residuals	28	9.3	0.33			

CR5000 (Δ*acr*B) - Tet - Kamba

	Df	Sum Sq	Mean Sq	F value	Pr(>F)	
H	1	4.4	4.42	0.996	0.3243	
AB	9	2654.6	294.95	66.415	<2e-16	***
H:AB	9	85.2	9.47	2.132	0.0491	*
Residuals	40	177.6	4.44			

CR5000 (Δ*acr*B) - Tet - Roundup

	Df	Sum Sq	Mean Sq	F value	Pr(>F)	
H	1	7.3	7.29	19.50	7.46e-05	***
AB	9	2826.5	314.06	840.02	< 2e-16	***
H:AB	9	36.7	4.07	10.89	2.50e-08	***
Residuals	40	15.0	0.37			

JW5503 ((Δ*tol*C) - Cip - Kamba

	Df	Sum Sq	Mean Sq	F value	Pr(>F)	
H	1	87.3	87.31	266.79	1.68e-14	***
AB	5	805.6	161.12	492.34	< 2e-16	***
H:AB	5	85.8	17.16	52.43	3.90e-12	***
Residuals	24	7.9	0.33			

JW5503 ((Δ*tol*C) - Cip - Roundup

	Df	Sum Sq	Mean Sq	F value	Pr(>F)	
H	1	3.2	3.16	9.961	0.00427	**
AB	5	1079.2	215.84	680.849	< 2e-16	***
H:AB	5	2.0	0.39	1.246	0.31921	
Residuals	24	7.6	0.32			

JW5503 ((Δ*tol*C) - Tet - Kamba

	Df	Sum Sq	Mean Sq	F value	Pr(>F)	
H	1	4.2	4.2	10.827	0.003083	**
AB	5	1990.0	398.0	1022.812	< 2e-16	***
H:AB	5	15.5	3.1	7.978	0.000151	***
Residuals	24	9.3	0.4			

JW5503 ((Δ*tol*C) - Tet - Roundup

	Df	Sum Sq	Mean Sq	F value	Pr(>F)	
H	1	6.9	6.9	224.4	1.12e-13	***
AB	5	2010.1	402.0	12991.0	< 2e-16	***
H:AB	5	27.1	5.4	174.9	< 2e-16	***
Residuals	24	0.7	0.0			

Signif. codes: 0 '***' 0.001 '**' 0.01 '*' 0.05 '.' 0.1 ' '

Abbreviations for variables:

H: herbicide concentration as a categorical variable
AB: antibiotic concentration as a categorical variable
H:AB: interaction term for both herbicide and antibiotic concentrations
T: treatment for Fig 1b and 2b (increase in herbicide concentration)

Supplementary Table 2: Minimum inhibitory concentrations of herbicide formulations for KEIO knock out strains

	BW25113 (WT)	JW0912 (Δ*omp*F)	JW5503 (Δ*tol*C)	JW2454 (Δ*acr*D)	CR5000 (Δ*acr*B)	CR7000 (Δ*acr*A)
Kamba	14500	14500	3000	12667	7333	7000
Roundup	4000	4000	100	4000	300	300

Herbicide concentrations are given in ppm ae.

Mar operon was upregulated by Kamba

The transcriptome of BW25113 was sequenced after culturing in only LB medium or medium supplemented with Tet and/or Kamba to further investigate the role of efflux pumps and OmpF (Supplementary Table 3). Genes that are known to be directly involved in adaptive resistance or their regulators were chosen for this analysis.

Of the monitored transcripts, only those of the *marRAB* operon significantly increased in supplemented media. The increase was larger for Kamba than for Tet, and was roughly additive when *E. coli* was exposed to both substances. This observation was consistent with another study that found an induction of *marRAB* upon exposure to salicylate [1]. Exposure to either or both substances only resulted in small and often statistically not significant changes in mRNA levels of the other genes selected. This observation was consistent with the change in transcription levels seen by others after exposure of *E. coli* GC4468 to 5mM Na-salicylate, the equivalent to our Kamba-only samples, or overexpression of *marA* [1]. Except for the *marRAB* operon, none of the genes we examined was identified as significantly up or down-regulated. With two exceptions, any changes in transcription levels in the Tet*Kamba samples could mostly be explained by the effect of Tet. These exceptions were *acrB*, which was moderately down-regulated by both Tet and Kamba individually, but moderately up-regulated in the Tet*Kamba samples, and *ompF*, which showed a non-significant increase with Tet, a small non-significant decrease with Kamba, but a larger and statistically significant decrease when exposed to both. The expression of the outer membrane porin OmpF is posttranscriptionally down-regulated by the antisense regulator *micF* [2], which could not be detected in the analysis due to its small size and the RNA isolation kit used. Small changes in *ompF*

transcription may not be reflective of protein levels. However, the direction of change is in agreement with the observation that deletion of *ompF* leads to increased tolerance in our growth experiments. While transcription levels changed for a number of transporters, no other member of the RND family was significantly increased, and *mdtE* and *mdtF*, components of the MdtEF-TolC efflux pump, were significantly decreased in all treatments. MdtEF-TolC is not known to be involved in Tet resistance [3]. However, a number of transporter genes of other families were up or down-regulated at the transcriptional level in the different treatments. There were a few cases where the combined treatment revealed a potentiation of the response to either Kamba or tetracycline. Examples for this were *yhiX*, coding for a predicted transporter of the MFS family [4]. Transcription increased non-significantly on exposure to either Kamba or Tet, but increased a significant 983.1-fold in the combined treatment. None of the three genes of the *cysAUW* sulfate/thiosulfate transporter responded to Kamba or Tet exposure by a significant change in transcription, but did for the combination (20.6, 9.9, and 6.9-fold increases, respectively). Similar up-regulation of this transporter has been shown in *S. enterica* sv. Typhimurium under acid stress conditions [5].

Supplementary Table 3: Change in the RNA expression level (multiples of RNA levels in LB) upon exposure of *E. coli* to Tet, Kamba, or Tet+Kamba.

	LB *vs* Tet	LB *vs* Kamba	LB *vs* Tet+Kamba
acrA	-1.11 (***)	1.12 (NS)	1.65 (***)
acrB	-1.11 (***)	-1.68 (***)	1.46 (*)
acrD	2.20 (*)	1.96 (NS)	2.18 (*)
marA	3.13 (***)	22.38 (***)	26.91 (***)
marB	4.11 (***)	17.06 (***)	29.27 (***)
marR	3.83 (***)	22.55 (***)	26.02 (***)
ompF	2.19 (NS)	-1.81 (NS)	-4.46 (*)
soxR	1.07 (NS)	-1.68 (NS)	1.07 (NS)
soxS	-2.35 (**)	1.16 (NS)	1.08 (NS)
tolC	-2.84 (***)	-1.25 (*)	-1.59 (***)

FDR corrected P-values are given in parenthesis. *, P<0.05; **, P<0.01; ***, P<0.001, NS, not significant.

Methods

A saturated culture of BW25113 was diluted 1:100 in LB and incubated at 37°C until OD_{600}=0.5. The culture was split and herbicides and/or antibiotics were added at the following concentrations: Kamba: 1380 ppm ae, Tet: 3 μg/mL. Three independent sub-cultures were grown for each condition (12 in total). Cultures were incubated for 1 hour before 10^8 cells were harvested, washed and resuspended in TE buffer. RNA was isolated using RNeasy Mini Kit (Qiagen, Valencia, CA, USA), DNase-treated (TURBO DNA-free kit, Ambion, Life Technologies, Carlsbad, CA, USA). PCR was used to confirm removal of DNA. rRNA was depleted using RiboZero magnetic kit (Illumina, San Diego, CA, USA), starting with 2 μg of total RNA. When necessary, RNA was concentrated using RNeasy MinElute Cleanup Kit (Qiagen, Valencia, CA, USA). Libraries were created using NEBNext Ultra Directional RNA library prep kit for Illumina (NEB, Ipswich, MA, USA) and NEBNext Multiplex Oligos for Illumina Index Primers Set. Samples were stored at -80°C. Concentrations of nucleic acid were confirmed at every stage using a NanoDrop spectrophotometer (Thermo Scientific, Wilmington, DE, USA). Library quality was evaluated using an Agilent Bioanalyzer and then sequenced by Tufts University Core Facility Genomics using an Illumina HiSeq 2500.

Statistics

Comparative transcriptome analyses used CLC Genomics Workbench 7.5.1 (https://www.qiagenbioinformatics.com) as adapted from others [6]. Prior to analysis, sequences representing 16S and 23S rRNA were removed. Sequences were mapped to the *E.coli* BW25113 genome (GenBank accession number CP009273), allowing mapping to intergenic regions, and with the following parameters and penalties (similarity fraction = 0.8, length fraction = 0.8, mismatch cost = 2, insertion cost = 3, deletion cost = 3). The RPKM (reads per thousand bases of a gene per million RNAseq reads) was calculated for each gene, and used to compare expression between experimental and control samples. Statistical analysis of these comparisons was performed with Baggerly et al.'s test [7] , and the Benjamini and Hochberg method was used to correct P-values for the false discovery rate [8].

References

1. **Pomposiello PJ, Bennik MH, Demple B**. Genome-wide transcriptional profiling of the *Escherichia coli* responses to superoxide stress and sodium salicylate. *J Bacteriol* 2001;183(13):3890-3902.
2. **Rosner JL, Chai TJ, Foulds J**. Regulation of ompF porin expression by salicylate in *Escherichia coli*. *J Bacteriol* 1991;173(18):5631-5638.
3. **Anes J, McCusker MP, Fanning S, Martins M**. The ins and outs of RND efflux pumps in *Escherichia coli*. *Front Microbiol* 2015;6:587.
4. **Szmidt-Middleton HL, Ouellet M, Adams PD, Keasling JD, Mukhopadhyay A**. Utilizing a highly responsive gene, yhjX, in E. coli based production of 1,4-butanediol. *Chem Eng Sci* 2013;103:68-73.
5. **Ryan D, Pati NB, Ojha UK, Padhi C, Ray S et al.** Global transcriptome and mutagenic analyses of the acid tolerance response of Salmonella enterica serovar Typhimurium. *Appl Environ Microbiol* 2015;81(23):8054-8065.
6. **Mortazavi A, Williams BA, McCue K, Schaeffer L, Wold B**. Mapping and quantifying mammalian transcriptomes by RNA-Seq. *Nat Methods* 2008;5(7):621-628.
7. **Baggerly KA, Deng L, Morris JS, Aldaz CM**. Differential expression in SAGE: accounting for normal between-library variation. 2003;19(12):1477-1483.
8. **Benjamini Y, Hochberg Y**. Controlling the false discovery rate: a practical and powerful approach to multiple testing. *J Royal Stat Soc* 1995;57:289-289.
